# Multiple familial cellular neurothekeomas: Report of 3 males with infantile onset

**DOI:** 10.1016/j.jdcr.2025.02.049

**Published:** 2025-04-11

**Authors:** Albert E. Zhou, Anusha Kambala, Michael John Murphy, Mary Wu Chang

**Affiliations:** a Department of Dermatology, University of Connecticut School of Medicine, Farmington, Connecticut; b George Washington School of Medicine and Health Sciences, Washington, District of Columbia; c Department of Pediatrics, University of Connecticut School of Medicine, Farmington, Connecticut

**Keywords:** autosomal dominant, familial, fibrohistiocytic tumor, nerve sheath tumor, neurothekeoma, pediatric, plastic surgery, skin tumor, syndrome

## Introduction

Cellular neurothekeoma is a rare, benign tumor of soft tissue long believed to originate from the peripheral nerve sheath but is now reclassified as a fibrohistiocytic tumor. The true pathogenesis remains uncertain. We present a family of three males with infantile onset of multiple cellular neurothekeomas, to our knowledge, represents the first report with familial association, notable for young age at presentation, and male sex.

## Case descriptions

### Patient 1

**Fig 1:**
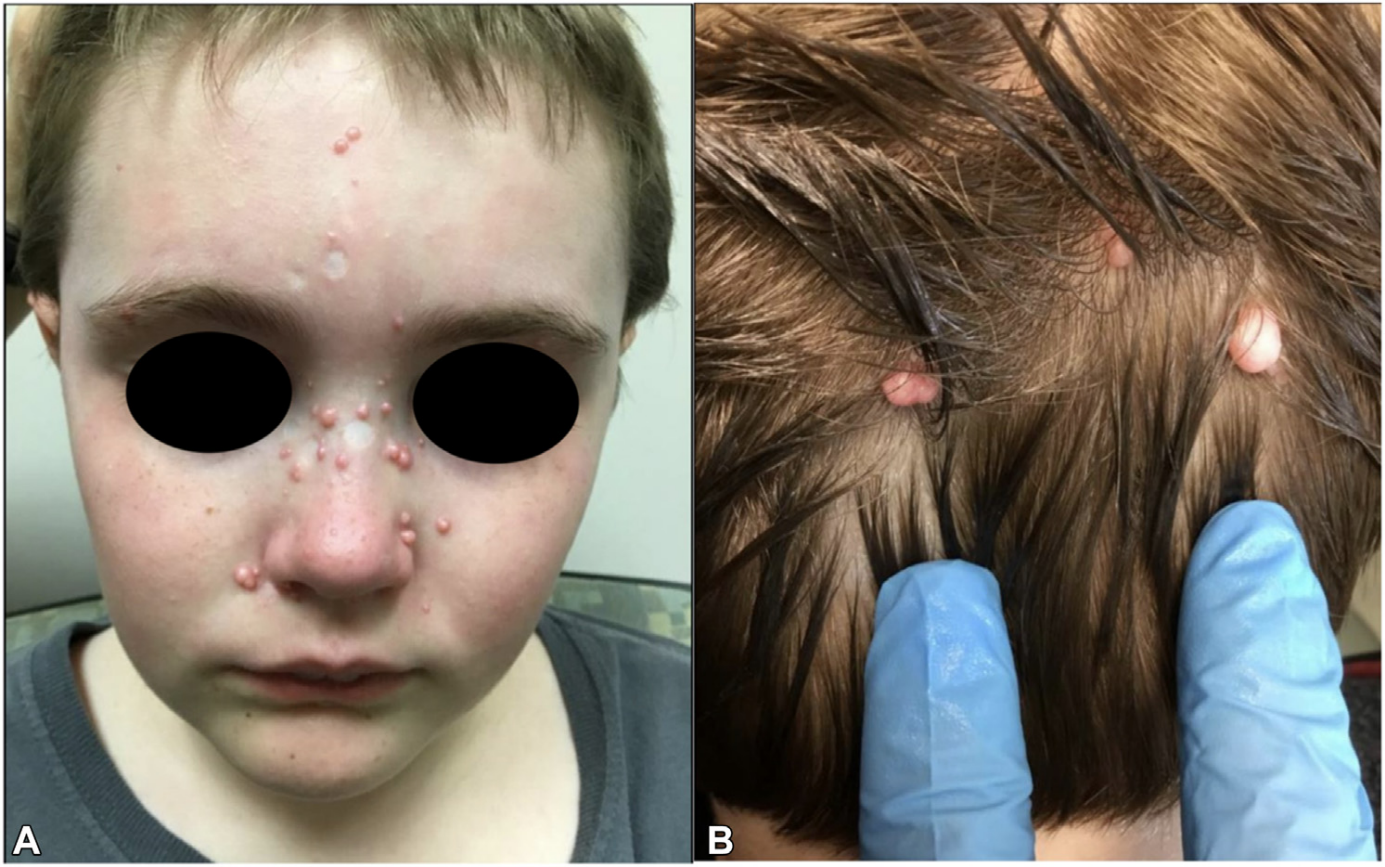
Patient 1 (proband), a 10-year-old man presenting with thirty 1 to 3 mm pink-to-skin-colored papules, others pedunculated, scattered throughout (**A**) bilateral cheeks, nasal dorsum, lower lips, forehead, and (**B**) occipital scalp. Circular scars are noted from previous shave biopsies/removals.

**Fig 2:**
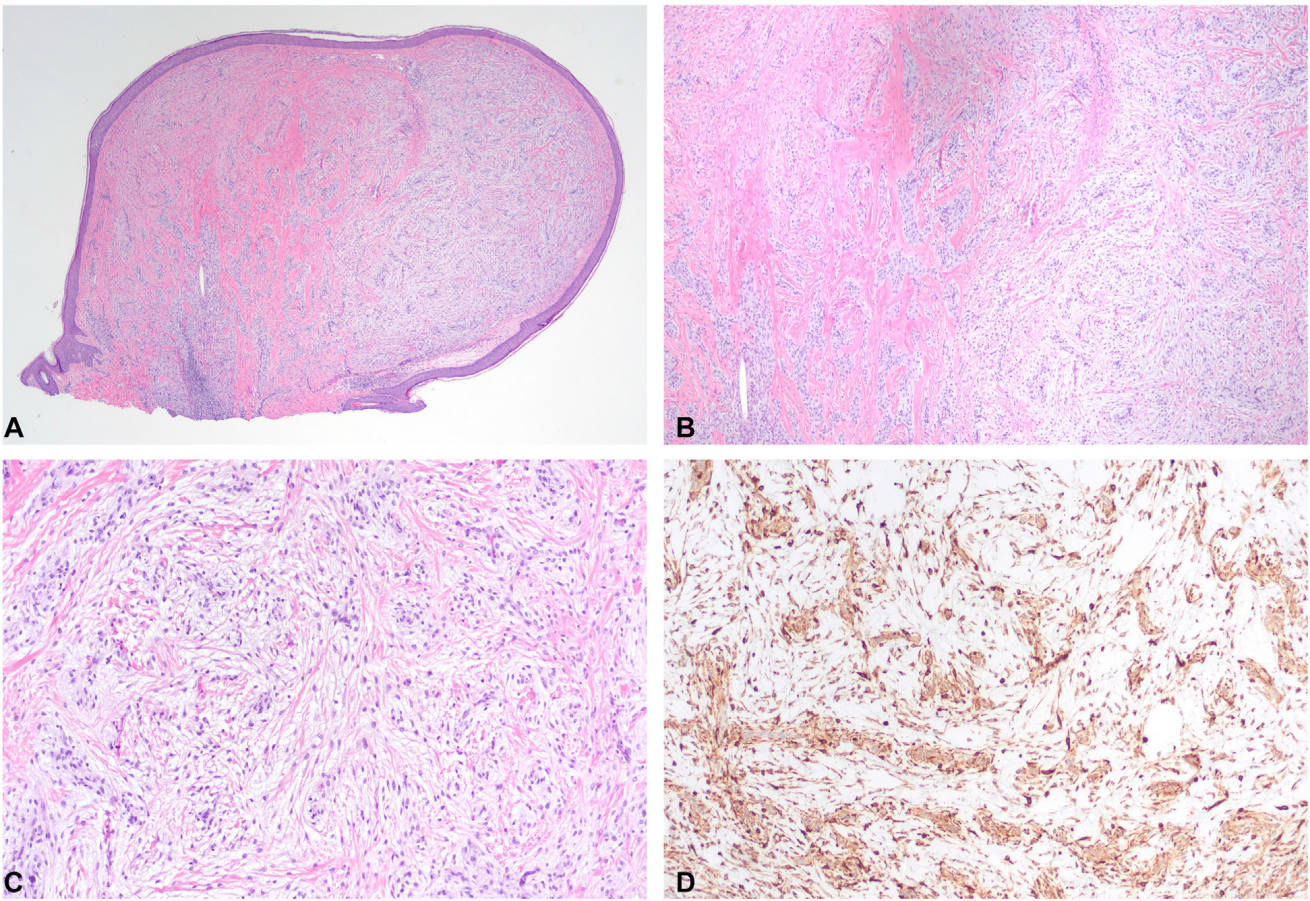
**A-D,** Histopathology of a biopsied scalp nodule from patient 1 demonstrated cellular neurothekeoma: nests and fascicles of pale cells within fibromyxoid stroma. Immunohistochemistry showed strong staining for NK1/C3 (**D**). (**A-C,** Hematoxylin-eosin stain; **D,** NK1/C3 stain; original magnifications: **A,** ×10; **B,** ×40; **C,** ×100.)

A 10-year-old boy presented with a 5-year history of numerous papulo-nodules on the face and back of the scalp. These had initially started when the patient was 9 to 10 months old, progressively increasing in size and quantity. These lesions were asymptomatic, without pain nor pruritus. Past medical history was notable for new-onset seizure during sleep at 6 years of age. An electroencephalogram indicated epileptogenicity from the bilateral independent centrotemporal regions, believed to be benign rolandic epilepsy. On examination, there were approximately 30 discrete, 1 to 3 mm, pink-to-skin-colored, smooth papules and nodules, some clustered, predominantly on the central face and nose, with additional nodules scattered on the occipital scalp (Fig 1, *A, B*). Darier sign was negative. There was no lymphadenopathy or organomegaly. A complete blood count and comprehensive metabolic panel were normal. He had multiple shave biopsies at 5 years of age, some leaving circular scars. Multiple biopsies from the original presentation, and more recent biopsy of a scalp nodule revealed similar findings: a dermal proliferation comprised of nests of histiocytoid/epithelioid cells with gray cytoplasm and indistinct borders, embedded in a fibromyxoid stroma (Fig 2, *A-C*). Immunostaining was positive for CD68, NK1/C3, and factor XIIIa, but negative for MART-1 or S-100 (Fig 2, *D*). A diagnosis of cellular neurothekeoma, polypoid-type was made. The patient was being bullied at school and sought removal of the facial lesions thus he was referred to Plastic Surgery. Five lesions were removed via elliptical excision with layered closure under general anesthesia, and the patient recovered uneventfully.

### Patient 2

**Fig 3:**
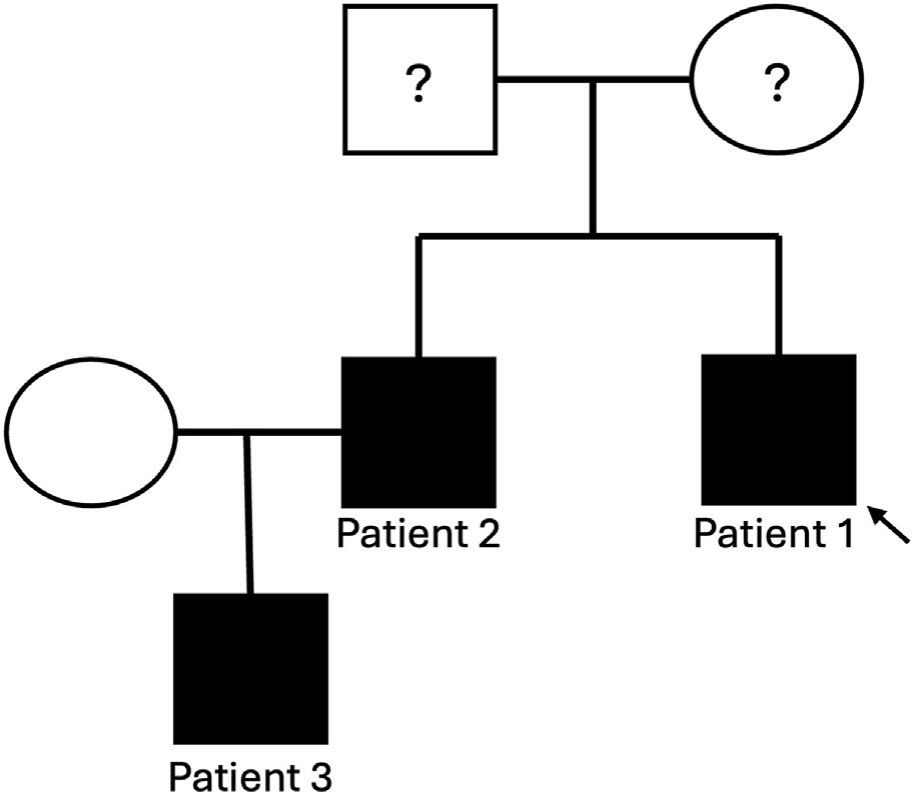
Pedigree of 3 family members with multiple neurothekeomas. Patient 1 is the proband. Assuming both of the proband’s parents are unaffected, possible inheritance patterns include: autosomal recessive; autosomal dominant inheritance with only male penetrance; or autosomal dominant but with gonadal mosaicism. Family declined genetic testing.

Patient 2, a 30-year-old man, was the older brother of patient 1 (Fig 3). At 2 years of age he developed facial papulo-nodules which then grew in size and quantity over time. Papulo-nodules also developed on the back of the scalp. These lesions have been asymptomatic without pain nor pruritus. The patient notes that any minor trauma to the lesions induces growth over the following days and lesions remain enlarged. Skin biopsy demonstrated the same light microscopic features and immunophenotype as patient 1.^1^ At 20 years of age, patient 2 had ablative CO_2_ laser therapy for the lesions on his face, which led to satisfactory clinical resolution. However, over the following years, new papulo-nodules have slowly reappeared in the nasolabial folds.

### Patient 3

**Fig 4:**
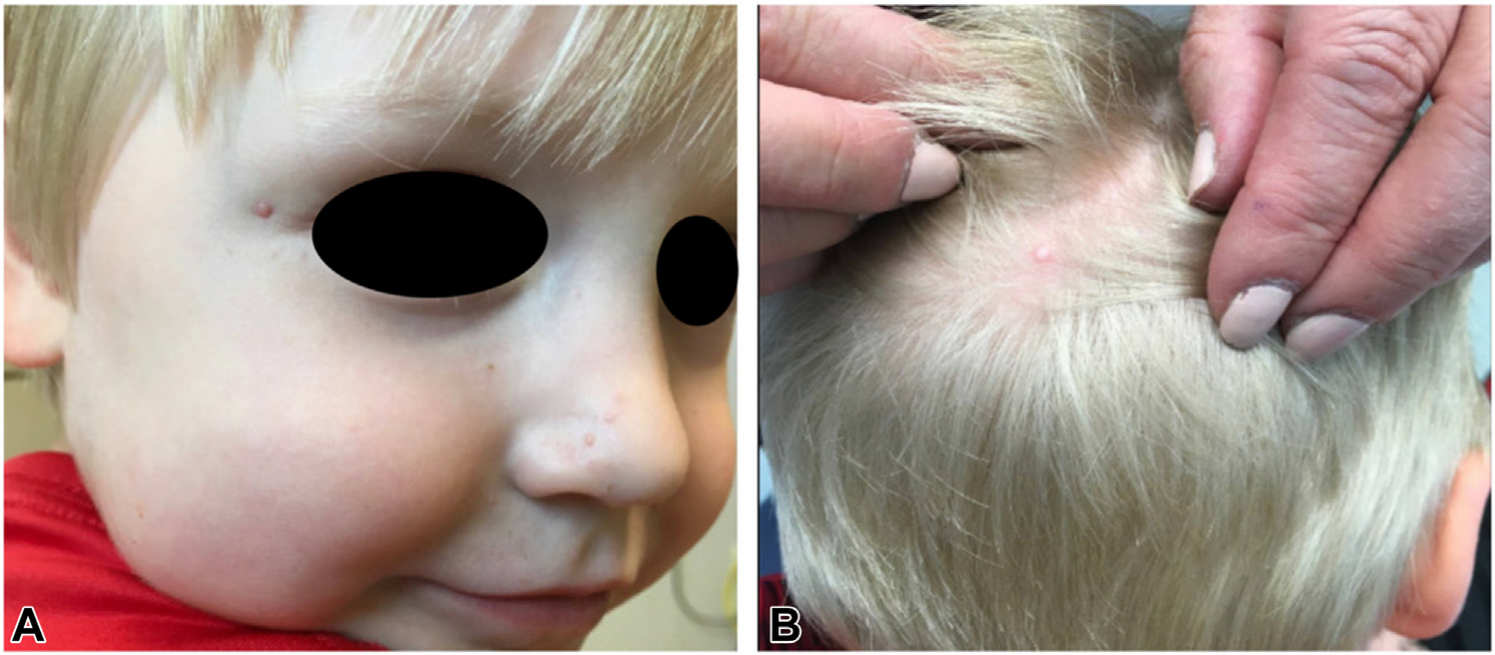
Patient 3, the 3-year-old son of patient 2, presented with multiple, tiny, nonspecific, tan-colored papules clustered on the central face, upper eyelids, and scalp (**A, B**). The lateral aspect of the right canthus shows the residual papule after spontaneous shedding of a pedunculated lesion (**A**).^2^

Patient 3 was the 3-year-old son of patient 2. At the age of 11 months, several pink, pedunculated papules appeared on his left eyebrow and lateral aspect of the right canthus and slowly grew (Fig 4). On examination, there were approximately 19 multiple, tiny, nonspecific tan-colored papules clustered on the central face, upper eyelids, and scalp. The largest lesion at the lateral aspect of the canthus was pedunculated and most of it spontaneously fell off. Shave biopsy of a scalp nodule demonstrated similar light microscopic findings as patients 1 and 2; stains were positive for NK1/C3 and negative for SOX-10 and S-100, consistent with cellular neurothekeoma.

## Discussion

Neurothekeoma is a benign tumor first described in 1969. The cellular variant was defined in 1986 and over 300 cases have since been reported^2^^,^^3^ pediatric cases are less common.^4^^,^^5^ Typically, cellular neurothekeomas present during the second and third decades of life, with a mean age of 21 years and 2:1 female to male predominance.^2^^,^^6^ Clinically, these lesions manifest as painless, slowly growing, solitary, reddish, dome-shaped nodules, most commonly located on the head, neck, and upper extremities.^7^ Despite their generally small size, ranging from 0.3 to 2.0 cm,^4^ there are cases where the lesions infiltrate into subcutaneous fat, skeletal muscle, or even surrounding vasculature.^6^

The primary treatment approach is complete surgical excision. Although there is no standardized protocol for surgical margins, grossly negative margins with a few millimeters of clearance are typically considered adequate.^6^ Although some authors recommend Mohs micrographic surgery, recurrences after simple excision are uncommon.

Cellular neurothekeoma should be included in the clinical differential diagnosis of cutaneous papules or nodules, especially in infants and children.^6^ Patient 1 was previously reported in an abstract describing multiple neurothekeoma mimicking molluscum contagiosum.^1^ Neurothekeomas can also mimic other entities such as adnexal neoplasms, or even cutaneous metastases.^1^ The differential diagnosis is broad: fibrous tumors (eg, dermatofibroma, dermatofibrosarcoma protuberans, and giant cell fibroblastoma), histiocytic tumors (eg, juvenile xanthogranuloma), lymphocytic tumors (eg, lymphoma), melanocytic tumors (eg, blue nevus, malignant melanoma, and Spitz nevus), muscle tumors (eg, leiomyoma), and neural tumors (eg, neuroblastoma and neurofibroma).^7^

Histologically, neurothekeomas are characterized by lobules of epithelioid and spindle cells embedded in a myxoid matrix, separated by fibrous connective tissue.^8^ Multinucleated cells can be found in all subtypes, and nuclear atypia with mitotic figures is common.^4^ The tumors usually exhibit poorly defined margins, which contributes to the challenge of complete surgical excision. Immunohistochemically, neurothekeomas stain positive for several markers, including S-100A6, NKI/C3, neuron-specific enolase, CD10, MITF, CD68, and α-smooth muscle actin, whereas being negative for S-100 protein, GFAP, Melan-A, and CD3.^6^ These findings help distinguish neurothekeoma from other soft tissue tumors.

Based on mucin content, cell density, growth pattern, and immunohistochemical profile, neurothekeomas can be categorized into 3 primary subtypes: myxoid, cellular, and mixed/intermediate.^2^^,^^9^ However, the terminology surrounding cellular neurothekeoma and nerve sheath myxoma (previously referred to as “myxoid neurothekeoma”) has been a source of confusion. Immunohistochemical analysis from 2 large case series has clarified that cellular neurothekeoma and nerve sheath myxoma are distinct entities, with nerve sheath myxoma staining positive for neural markers such as S-100, whereas cellular neurothekeoma stains positive for fibrohistiocytic markers.^2^^,^^9^

Although genetic studies on neurothekeoma are limited, emerging evidence suggests potential genetic underpinnings. Multiple cellular neurothekeomas may be associated with specific syndromes,^10^^,^^11^ including Guillain-Barré syndrome,^12^ Birt-Hogg-Dubé syndrome,^13^ and increased estrogen production.^14^ However, a recent review of 6 patients with multiple neurothekeomas found no consistent genetic link.^7^ A recent microarray analysis of 9 cellular neurothekeomas revealed upregulation of stromal glycoproteins and metalloproteinases, proteins involved in mesenchymal differentiation, further supporting the hypothesis of a fibrohistiocytic origin.^8^ Additionally, similar expression of PGP9.5 in cellular neurothekeoma and fibroblastic lesions lends further evidence to the theory of fibroblastic differentiation.^15^

Genetic mutations have also been identified in individual cases. Next-generation sequencing of a neurothekeoma from a 53-year-old man revealed 4 specific point mutations: PI3K w552∗, ALK P1469S, SMO G461S, and ERBB3 L77M.^16^ Aberrant expression of TFE3, a transcription factor involved in lysosomal biogenesis and immune response, has been reported in a case series, indicating its potential role in the pathogenesis of cellular neurothekeoma.^17^ Together, these findings suggest a genetic basis for cellular neurothekeoma, particularly in familial cases.

This is the first report, to our knowledge, of familial inheritance of cellular neurothekeomas. In addition, it is unusual in that all 3 patients were male, and the onset was in infancy (9 months, 11 months, and 2 years of age). In contrast, neurothekeoma usually presents in adults, commonly females in 2nd the 3rd decades of life. The face and scalp location, and clustered presentation was consistent with the usual distribution of multiple neurothekeomas. Unfortunately, the family has declined genetic testing. Given the lack of phenotypic information from the proband’s parents, several inheritance patterns remain possible (Fig 3): these include autosomal recessive inheritance, assuming the proband’s mother (wife of patient 2) is a carrier; autosomal dominant inheritance with male-limited penetrance; or autosomal dominant inheritance with gonadal mosaicism. The possibility of increased risk of other proliferative neoplasms in individuals with this genetic variant warrants further exploration. Additional reports of familial cases of neurothekeoma will be invaluable in elucidating the genetic and clinical characteristics of this rare tumor.

## Conflicts of interest

None disclosed.
